# Theta Oscillations, Oculomotor Processing, and Neural Synchronization: A Review

**DOI:** 10.3390/brainsci16060555

**Published:** 2026-05-22

**Authors:** Chiagoziem Anigbogu, Matthew N. Svalina, Gavin R. Hoffman, Aditya Kumar, Kevin Tyner, John A. Thompson, Daniel R. Kramer

**Affiliations:** 1 Department of Neurosurgery, University of Colorado Anschutz Medical Campus, Aurora, CO 80045, USA; 2 School of Medicine, University of Colorado Anschutz Medical Campus, Aurora, CO 80045, USA

**Keywords:** theta oscillations, oculomotor processing, memory, neural synchrony, SEMs, movement disorders, epilepsy, Parkinson disease, Alzheimer disease

## Abstract

**Highlights:**

**What are the main findings?**

- Saccadic eye movements (SEMs) and theta oscillations are tightly coupled, with SEMs inducing phase resets that synchronize cortical, hippocampal, and oculomotor networks.
- Theta oscillations act as an intrinsic timing mechanism that gates the initiation, direction, and efficiency of eye movements during visual exploration and memory processing.

**What are the implications of the main findings?**

- Disruption of theta oculomotor coupling represents a shared mechanism underlying cognitive deficits in neurological disorders such as Parkinson disease, Alzheimer disease, and epilepsy.
- Theta oculomotor dynamics provide a promising framework for developing noninvasive biomarkers and targeted neuromodulation strategies to improve cognition and memory.

**Abstract:**

Theta oscillations, neural activity within the 4–8 Hz range, are implicated in a wide range of cognitive functions, including oculomotor and sensory processing, attention, memory, and motor planning and execution across diverse brain regions. Saccadic eye movements (SEMs), which are integral to visual perception and cognition, occur within a similar frequency range. This review explores how theta oscillations contribute to oculomotor and cognitive processing, emphasizing their role in coordinated motor and sensory functions. We synthesize foundational and contemporary studies into a working model describing neural synchronization across cognitive networks. We discuss the complex interplay between theta oscillations, SEMs, and cognition, summarizing the current state of our knowledge.

## 1. Introduction

Theta oscillations, rhythmic neural activity in the ~4–8 Hz range, were first formally described during subcortical recordings in the hippocampus of rabbits following nociceptive stimuli by Jung and Kornmüller in 1938 [1]. Subsequent seminal work by Landfield et al. formally linked theta oscillations to memory formation in 1972 [2]. Since that time, theta oscillations have been implicated in a wide array of diverse cognitive processes including, sensation and perception [3,4,5,6,7], attention [8,9], working memory [10,11,12], spatial navigation [13], motor planning [14,15], and cognitive control [16,17]. Perhaps the most extensively understood role for theta oscillations is in memory processing [18,19,20,21]. Indeed, theta oscillations in the hippocampus and their role in synchronization amongst distinct cortical networks is thought to be integral for successful memory encoding and retrieval [22,23,24,25,26,27,28]. Theta oscillations are now understood as part of the fundamental mechanism for coordinating neural activity across distributed networks including the oculomotor system [29,30,31], prefrontal and frontal cortices [16,25], medial temporal lobe (MTL) [21], parietal lobe [32], and amygdala [33].

Saccadic eye movements (SEMs), rapid ballistic eye movements that shift the fovea from one point of fixation to another, occur at a frequency of ~3–5 Hz rate [34,35,36]. Similar to theta oscillations, SEMs have been shown to play a role in attention [37], working memory [38], and memory encoding and retrieval [39]. Further, SEMs are known to modulate theta oscillations [40,41,42]. For instance, SEMs have been shown to reset theta phase during memory-guided eye movements [30,43,44]. Indeed, growing evidence demonstrates that SEMs and theta oscillations are intrinsically and dynamically linked [45,46]. These studies suggest a dynamic and bidirectional relationship between oculomotor processing, oscillatory neural activity, and memory encoding [47,48,49,50,51,52,53,54].

This review examines the current state of our understanding of how oculomotor processes, particularly SEMs and fixation events, influence cognition including visual processing and memory. Through synthesis of both human and animal studies, we discuss evidence that SEMs both shape and are shaped by oscillatory neural activity. We highlight evidence for a bidirectional dynamic interaction between SEMs and theta oscillations and discuss important mechanisms underpinning this relationship, including phase-reset, theta-theta coherence, cross-frequency coupling, and corollary discharge (CD). We conclude by discussing the translational relevance of these findings for neurological disorders and highlight possibilities for novel diagnostic and therapeutic strategies.

A focused literature review was performed using PubMed, Embase, and Google Scholar to identify studies relevant to the influences of saccadic eye movements and oculomotor processes on cognition, learning, and memory, with particular emphasis on electrophysiological correlates such as theta oscillations measured using intracranial electroencephalography (iEEG/ECoG), electroencephalography (EEG), and related recording modalities. Search terms included, but not limited to, combinations of “saccadic eye movements,” “theta oscillation,” “phase-reset,” “memory,” “learning,” and “cross-frequency coupling.” The search included articles through 2025. Titles and abstracts were screened for relevance, followed by a full-text review when appropriate. Priority was given to prospective human and non-human primate studies, seminal publications, and recent systematic reviews. Reference lists of included studies were screened to identify additional relevant publications.

## 2. Oculomotor Behavior Entrains Cortical and Subcortical Oscillations

SEMs are rapid ballistic movements of the eyes that redirect the fovea to areas of interest within the visual field. Typical SEMs velocities range from 30 to 500° of angular rotation per second, with durations of approximately 20 to 200 milliseconds [36]. This translates to a frequency of 3–5 Hz, which is approximately the frequency at which theta oscillations occur. SEMs enable rapid sampling of visual scenes with successive SEM-fixation cycles constructing detailed representations of the environment and forming memory associations for foveated objects, their spatial arrangement, and context [55,56,57].

Between SEMs, fixations occur with the eyes remaining still for variable lengths of time of up to 200 to 300 milliseconds. Interestingly, visual input is thought to be transiently suppressed during SEMs, reducing the perception of low-quality visual information [57,58,59]. Thus, SEMs allow rapid sampling of visual fields, whereas fixations allow for the detailed processing of focused visual information. Together, SEMs and fixations form the oculomotor basis of active sensing and visual exploration [48,60,61]. The oculomotor system, which controls these movements, includes several brain regions including the superior colliculus (SC), the frontal eye fields (FEF), and the parietal association cortex. These distinct anatomical regions coordinate the initiation, direction, and amplitude of SEMs, ensuring precise and efficient eye movements [62,63,64,65,66]. Notably, the frequency of SEMs temporally aligns with the frequency of theta oscillations, suggesting potential coordination and interaction between the two.

Numerous studies have provided direct evidence that SEMs synchronize cortical and mesiotemporal networks. In macaques freely viewing natural images, SEMs were found to occur at a frequency of ~3–4 Hz, periodically entraining the phase of low-frequency local field potentials (LFPs) in V1 and V4 and increasing concurrent gamma-band synchronization. It is thought that this high-frequency activity reflects local processing. Thus, this theta-paced rhythm has been proposed to act as an internal timing signal that aligns high-frequency visual processing with oculomotor sampling, providing correlative evidence consistent with tight coupling between oculomotor movements and cortical oscillations [48]. In V1 in the absence of visual input, fixation onset triggers a phase-aligned theta transient followed by an increase in gamma-band power. Thus, fixation onset alone is associated with theta phase reset and increases in gamma-band activity during visual exploration, consistent with a model in which an extraretinal CD, an internal efferent copy of a motor program which informs sensory processing, may prime visual cortex for the next input cycle [67]. Further, laminar recordings from V1 show that SEMs are accompanied by theta phase realignment across all layers and sharpened supragranular gamma bursts, suggesting that the oculomotor CD may temporally organize visual-cortical excitability during active sensing [68]. Parallel studies in primate primary auditory cortex (A1) reveals a similar cross-modal mechanism, with SEM-locked theta phase resets observed in the extragranular laminae of A1 [68,69].

Within frontoparietal circuits, sub-threshold microstimulation of the FEF entrains an 8–12 Hz oscillation that biases the latency and direction of ensuing SEMs and selectively enhances gamma power in retinotopically corresponding sites of lateral intraparietal cortex (LIP), providing evidence that frontoparietal alpha can gate attentional selection upstream of the SC [70]. Concurrently, theta-driven spike-field coherence in the anterior cingulate cortex (ACC) phase-locks with FEF LFPs, and the strength of this coherence is associated with accurate sensorimotor mapping during memory-guided SEMs [49].

Further, mesiotemporal structures also exhibit robust SEM-driven phase resets. Interestingly, this phase alignment is task-dependent. Hippocampal recordings in both humans and macaques recorded during free viewing showed that SEMs realign 3–8 Hz LFPs within ~200 ms of fixation onset. These data are consistent with a conserved SEM-locked theta phase realignment in hippocampal networks across species [43]. Similarly, SEMs directed toward socially salient human faces, compared to nonsocial objects, selectively enhanced theta-theta coupling between hippocampus and amygdala. These data suggest that SEM-aligned phase coherence may index flexible engagement of memory networks according to behavioral context [71].

Similarly, hippocampal LFPs in both macaques and humans show that SEMs during free exploration time-lock a 3–8 Hz rhythm within ~200 ms of fixation onset, independent of SEM mechanics, realigning the hippocampal theta cycle to each new fixation [43]. In macaque hippocampus, this saccade-triggered hippocampal phase reset and the magnitude of pre-fixation theta power predicts improved recognition performance [44]. In human hippocampal intracranial recordings, fixations aligned with distinct theta phases (i.e., peak versus trough) are differentially linked to memory retrieval and encoding [30]. Together, these studies are consistent with a model in which intrinsic theta oscillations are recruited to time SEMs and fixations in ways that may optimize memory processing. Thus, the oculomotor system appears to rhythmically co-vary with cortical and subcortical processing through low-frequency phase realignment, gamma synchrony, and context- and behavior-dependent network engagement. SEMs may therefore represent an important link between active vision and memory encoding and retrieval [72].

## 3. Oscillatory Dynamics Gate and Modulate Oculomotor Movements

Converging evidence suggests that low-frequency cortical oscillations are not only modulated by SEMs but may also serve as an internal metronome for the temporal coordination of oculomotor processing [73,74]. Recent studies in both humans and non-human primates (NHPs) have shown that the phase of cortical oscillations influences SEM onset, direction, and latency. For example, simultaneously recorded LFPs from the FEF and V4 in rhesus monkeys demonstrated that SEMs were strongly phase-locked to 4–8 Hz theta oscillations in these brain regions. Interestingly, theta-phase alignment selectively increased the likelihood of SEMs directed away from a visual target [75].

Throughout this review, we use “theta” to refer to the canonical 4–8 Hz range, “alpha” to ~8–12 Hz, and “low-beta” to ~13–22 Hz. The literature on oscillatory gating of oculomotor behavior spans all three bands. Where studies report effects outside the canonical theta range, we identify the relevant band explicitly. These bands likely reflect partially distinct mechanisms. Theta is most consistently associated with hippocampal-mediated memory encoding and exploratory sampling, whereas alpha/low-beta in frontoparietal circuits is more often linked to attentional gating and motor selection (though the boundaries are not always sharp) and a unified account of how these adjacent rhythms relate to one another in the oculomotor system remains an open question.

Complementary studies in humans similarly showed that the phase of low frequency oscillations immediately preceding target presentation predicts SEM reaction time, though notably in a band adjacent to theta. Specifically, the phase of ongoing high alpha/low-beta (~11–17 Hz) oscillations before target onset predicted reaction time with SEMs initiated faster when the stimulus onset was coincident with an optimal phase angle. By jittering target-stimulus location and timing and analyzing phase-behavior relationships, SEMs triggered at defined phase angles during oscillatory activity showed shorter latencies, whereas those triggered half a cycle later exhibited longer latencies. Thus, oscillatory activity, both within and outside of the theta band, may act as an endogenous clock correlated with the timing of oculomotor activity, with phase potentially constraining when a SEM is initiated [73]. Interestingly, it is plausible that low-frequency oscillations beyond the theta band may also encode information driving directionality in SEMs. Indeed, a recent study in rhesus monkeys performing a visual foraging task demonstrated that SEMs are tightly phase locked to alpha/low-beta oscillations (~9–22 Hz) in the FEF, with the specific phase of these oscillations predicting SEM direction 100 ms before the SEM was initiated. Taken together, these data suggest that frontoparietal alpha/low-beta activity, rather than theta specifically, may modulate the competitive dynamics of target selection [74].

During natural reading in healthy human subjects, SEMs occur at ~4–5 Hz followed by ~200–250 ms fixations. The sequence of word-to-word SEMs was found to be phase-locked with theta-band EEG activity in the absence of any external pacing [76]. Thus, oculomotor processing occurs at an intrinsic rhythm coherent with network theta activity, suggesting that neural synchronization functions as an internal pacemaker timing oculomotor sampling. Endogenous theta rhythmicity may gate visual sampling by precisely timing SEMs and fixation events to be coherent with theta oscillations to enhance memory encoding and processing. Consistent with this, studies have noted that theta oscillations in fronto-parietal networks correlate with shifts in attention and that the SEM-fixation cycle may reflect an endogenously timed sampling mechanism [4,77,78,79,80,81,82]. Together, these data indicate that theta oscillations align active visual exploration to optimize neural networks for memory encoding [44].

Similarly, sub-threshold microstimulation in macaque FEF during the delay of a visual search task selectively increased gamma-band power in retinotopically-matched LIP neurons. Interestingly, this suggests that oscillatory activity modulation may bias attentional selection and SEM direction without altering latency [70].

Direct manipulations across species further support this role. Optogenetic stimulation at theta-band frequencies (5–10 Hz) in the SC of rodents has been shown to entrain contralateral saccade-like head-orienting movements [83]. The kinematics of these head-orienting movements resemble the sequential saccades elicited by stimulation of the SC in NHPs [84]. Thus, induction of oscillatory patterns in oculomotor circuits can bias SEM onset, latency, and direction. Theta-band stimulation appears particularly effective at synchronizing hippocampal and midbrain circuits for SEM timing [83], whereas alpha/low-beta phase has been more consistently implicated in frontoparietal target selection [70].

Further, direct manipulations in humans have also provided converging evidence. In healthy adults, applying a 5 Hz stimulation frequency using transcranial alternating-current stimulation (tACS) over the FEF during natural reading increased fixation frequency to precisely match the externally applied rhythm, reduced fixation dwell time, and decreased total reading time. Although SEM kinematics were not fully characterized, it stands to reason that the external induction of theta oscillatory activity within the oculomotor system may facilitate the SEM-fixation cycle. Overall, in humans, the evidence that theta oscillatory activity directly drives behavior remains indirect. Rigorous and direct manipulations are needed to firmly establish a direct role for oscillatory activity in driving SEMs [85].

## 4. Network Architecture in Oculomotor Processing

**Figure 1:**
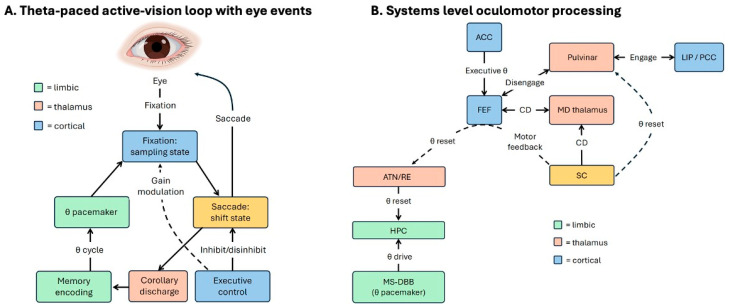
Proposed Model for Theta and Oculomotor Movement. (**A**) This diagram shows a proposal of how fixation and events alternate and are embedded within hippocampal theta (θ) cycles in visual exploration and memory formation. During fixation, cortical gain is maximized, allowing detailed sensory processing aligned with theta activity to support encoding of memory. This process is paced by continuous theta cycles. The anterior cingulate cortex and frontal eye field govern the transition to s by providing inhibitory or permissive signals that initiate gaze shifts. Each in turn generates corollary discharge signals; these signals reset the cortical and hippocampal theta phases, preparing the system for the next fixation. The bidirectional nature of this loop illustrates how theta rhythms not only affect timing but are also reset by them, coupling oculomotor sampling with the temporal structure of memory processing. The color coding denotes limbic (green), thalamic (orange), and cortical (blue) components. Solid arrows indicate direct signaling pathways, while dashed arrows indicate modulatory or feedback signals. (**B**) This diagram suggests the networks that join saccadic eye movements and theta (θ) oscillations. A general proposal for the structural and functional control of eye movements and rhythmic activity is as follows. The frontal eye field (FEF) is the hub that integrates theta input from the anterior cingulate cortex (ACC), corollary discharge (CD) signals from the mediodorsal (MD) thalamus, and motor feedback from the superior colliculus (SC). The SC initiates s and drives CD that aligns cortical activity with upcoming eye movements. The pulvinar provides attentional gating, relaying signals to the lateral intraparietal cortex/posterior cingulate cortex (LIP/PCC) to engage visuospatial processing. In parallel, the anterior thalamic nucleus/nucleus reuniens of the thalamus (ATN/RE) synchronizes hippocampal (HPC) activity via theta reset, aligning these rhythms with input from the medial septum–diagonal band of Broca (MS-DBB), the primary theta pacemaker. Together, these cortico-thalamic-limbic circuits establish a closed-loop system in which saccadic initiation, CD, and theta phase resets ensure stable perception, attentional control, and memory encoding during visuospatial processing. The color coding denotes limbic (green), thalamic (orange), and cortical (blue) components. ACC = anterior cingulate cortex; ATN = anterior thalamic nuclei; CD = corollary discharge; FEF = frontal eye fields; HPC = hippocampus; LIP = lateral intraparietal area (posterior parietal cortex); MD thalamus = mediodorsal thalamus; MS-DBB = medial septum–diagonal band of Broca; PCC = posterior cingulate cortex; Pulvinar = pulvinar nucleus of the thalamus; RE = nucleus reuniens of the thalamus; SC = superior colliculus; Executive θ = midfrontal/ACC theta associated with cognitive control; Motor Feedback = motor feedback (primarily CD conveyed from SC via MD); Inhibit/Disinhibit = executive signals that suppress or permit saccade initiation.

Active vision involves both covert sampling involving attentional shifts without SEMs and overt sampling via SEMs. These complementary behaviors are orchestrated by intrinsic oscillatory rhythms distributed across cortical and subcortical networks [43,86,87]. In macaque visual cortex, a ~4 Hz theta rhythm synchronizes activity between V1–V2 with V4. During each theta cycle, gamma power rises during the high-gain phase of the theta cycle and falls at the opposite phase, demonstrating phase-amplitude coupling consistent with cyclic modulation of visual input [88]. Indeed, the enhancement of V4 responses produced by covert attention emerges only after a SEM directed toward the attended target [86]. These findings support a model in which vision proceeds through alternating sampling and shift phases, with cortical and subcortical activity aligning to theta phase in ways that may optimize encoding (Figure 1A) [87]. Importantly, the timing of SEMs is coupled to low-frequency oscillatory activity. In macaques engaged in naturalistic foraging or free-viewing tasks, SEMs are phase-locked to specific phases of LFP oscillations in the FEF, particularly within the alpha/low-beta range (9–22 Hz). The pre-saccadic phase of these oscillations can predict both onset and direction of the subsequent SEM [74]. This phase-locking illustrates how overt attention involving SEMs is precisely gated by frontoparietal alpha/low-beta rhythms, which may operate alongside (rather than as part of) the hippocampal and midbrain theta dynamics described above. Whether these adjacent bands reflect a single cross-frequency mechanism or distinct circuits operating in parallel remains in question. Similarly, during free-gaze visual-search tasks, SEMs are phase-locked to the theta cycle in both FEF and visual area V4, with coherence strength dependent on SEM direction [75]. Simultaneous recordings from FEF and LIP demonstrate that these fluctuations are linked to theta oscillations in the frontoparietal attention network [89]. In parallel, human behavioral studies show rhythmic cycles of enhanced and diminished visual-target detection following attentional cues consistent with theta-mediated sampling of visual information [80]. In sum, these findings indicate that SEMs and theta-band oscillatory activity are deeply intertwined across widespread cortical and subcortical networks, providing a rhythmic temporal scaffold for visual exploration [44,87].

## 5. Executive Function in Cognitive Control

Cognitive control over saccades involves suppressing reflexive glances in an anti-saccade paradigm or maintaining a target in working memory. This behavior engages a midfrontal network involving the anterior cingulate cortex (ACC) and FEF. In macaques, the ACC and FEF exhibit robust 3–9 Hz coherence during the delay before memory-guided or anti-saccade with the ACC leading the FEF in phase. Indeed, phase coherence between ACC–FEF predicts performance accuracy [49]. Similarly, studies in humans in a cued anti task have also demonstrated engagement of midfrontal and frontoparietal networks, with theta coherence increasing during preparation for correct anti- versus pro- behavior [90]. Human intracranial recordings extend these observations, showing that deliberate SEM choices are preceded by a long-range 5–10 Hz network linking the medial prefrontal cortex and parietal cortex, with the medial prefrontal node leading in phase [91]. Finally, when spatial attention is engaged, FEF spiking activity is phase locked to theta in the LIP. The strength of phase-locking predicts faster visual detection [92]. Taken together, these findings are consistent with a role for theta oscillations in coordinating activity across ACC, FEF, and LIP nodes during executive control of oculomotor behavior, with the timing of saccade suppression, selection, and release potentially organized by these rhythms.

## 6. Theta-Phase Coordination Between the Hippocampus and Oculomotor System During Visual Exploration

Beyond frontoparietal circuits, hippocampal–oculomotor interactions play a key role in linking eye movements to memory [93,94,95]. As previously described, intracranial recordings in humans and NHPs demonstrate that during free viewing and visual-search tasks, hippocampal LFPs exhibit a robust 3–8 Hz theta phase that resets and phase-aligns with each SEM-fixation cycle. Interestingly, the strength of this phase reset is novelty dependent [43,44,75]. This phase alignment is also task-dependent with evidence demonstrating that a novel scene search can induce stronger hippocampal theta phase alignment than viewing familiar images. Notably, while hippocampal theta phase reset is centered on SEMs, no accompanying increase in theta-band power is observed, indicating that SEMs may reflect a precise re-timing mechanism. Thus, transient phase locking of hippocampal theta with oculomotor movements is consistent with a model in which each SEM and fixation is timed to ongoing theta cycles, potentially supporting the encoding of visual information into memory. Consistent with this, hippocampal theta rhythms have been found to correlate with memory-guided viewing behavior. Intracranial recordings from neurosurgical patients performing a visual-memory task demonstrate that the pre-fixation phase of 4–6 Hz hippocampal theta reliably distinguished SEMs guided by memory from those directed toward novel targets. Thus, theta phase biases gaze according to memory processing demands [30]. Specifically, fixations centered on a familiar location were preceded by a peak in 4–6 Hz hippocampal theta phase, whereas fixations centered on a novel location followed the trough of the theta cycle, revealing a phase-dependent separation of memory-guided and novelty-driven SEMs. Taken together, this evidence is consistent with a model in which hippocampal theta phase is associated with the timing of SEMs toward novel versus familiar targets, potentially modulating downstream oculomotor circuits in a context-dependent manner. Moreover, cross-frequency analyses demonstrates that the hippocampal theta phase can organize high-frequency gamma bursts differentially for retrieval vs. novel input, with retrieval-linked SEMs initiating at a specific theta phase with novelty detection at the opposite phase. Together, these findings describe a hippocampal-cortical mechanism whereby theta oscillations align with oculomotor sampling of visual stimuli, thereby coupling memory processing to the active sensing of the environment.

## 7. Subcortical Relays Coordinate to the Cortical-Theta-Saccade Loop

The SC is a laminated midbrain structure with a well-known role in the initiation of SEMs [84]. In rhesus macaques, the SC has also been shown to initiate a CD signal through the mediodorsal (MD) thalamus to the FEF. This CD signal time-locks FEF activity to SEMs. Disruption of the thalamic relay abolishes the timing alignment and undermines post-SEM visual stability [96,97]. In humans, intracranial recordings reveal that an SEM-linked CD signal reaches the hippocampal formation and midline thalamic nucleus reuniens, transiently suppressing broad-spiking neurons, exciting narrow-spiking neurons, and resetting local theta phase just before each eye movement [98]. Simultaneous recordings from pulvinar, FEF, and LIP show that the pulvinar shifts alpha/low-beta coherence between the two cortical networks according to the ongoing theta phase, dynamically routing information flow during “engage” versus “disengage” epochs [99,100]. Cue onset resets theta phase throughout the cortical column, and deep-layer neurons in both FEF and LIP spike at a consistent theta phase, implying that the motor drive relayed to the SC is released on a theta-paced schedule [89]. Taken together, these data suggest that combined with thalamic and cortical mechanisms, these subregional circuits transiently align frontal, parietal, thalamic, and hippocampal networks in a common rhythm, consistent with a role for SEMs in jointly coordinating perception, action, and memory encoding (Figure 1B).

The framework we propose here brings together several lines of evidence into a single account. Prior work has shown that SEMs and fixations reset low-frequency oscillations during active vision [68,72,101], that frontoparietal theta rhythms organize attention into alternating sampling and shifting states [43,89,102,103], that hippocampal theta phase separates memory retrieval from novelty processing [18,30], and that SEMs carry CD signals into memory-related structures [98]. These findings are consistent with a model in which SEMs, fixations, and CD work together to align low-frequency activity across visual, oculomotor, thalamic, and hippocampal circuits.

A key feature of this model is its temporal asymmetry. Specifically, theta phase before a SEM may bias where the eyes move next on the basis of memory, whereas phase reset after fixation may support the encoding of new information or the updating of existing representations. This framework also generates several testable predictions. Pre-saccadic theta phase should differ for memory-guided versus novelty-oriented SEMs. Fixations on novel images should elicit stronger post-fixation theta-gamma coupling than fixations on familiar images. In addition, disrupting saccade-related relay signals should preferentially impair memory-guided SEMs relative to novelty-driven orienting. An important complement to this framework comes from recent work on rhythmic attentional sampling, which suggests that attention can fluctuate rhythmically even in the absence of overt eye movements [87,104]. In particular, covert spatial attention and its neural signatures do not always depend on microsaccades, suggesting that attention and oculomotor control are tightly linked but can operate independently.

## 8. Clinical Implications and Future Directions

In the healthy human brain, SEMs and theta oscillations are tightly coupled. Each SEM or fixation enables a phase reset that synchronizes oscillatory activity and oculomotor movements to effectively process incoming visual information [74,86]. This timing likely underpins the oscillatory gating of perception and memory encoding [102]. Importantly, this oculomotor entrainment is not limited to the theta band. Interestingly, in the absence of visual input, SEMs alone trigger broadband spectral transients that propagate from occipital cortex toward mesial temporal regions demonstrating that an SEM alone can phase reset and drive activity across the visual–memory network [67,68]. For instance, during memory encoding, SEMs are phase-locked to alpha band activity in occipital and MTL regions [105]. These observations suggest that in healthy individuals the oculomotor and cognitive systems flexibly couple to oscillatory frequencies that best supports the ongoing behavior (i.e., theta for exploratory sampling and spatial memory, alpha for fluent reading). Functionally, the oculomotor system supplies an internal timing pulse that aligns cortical oscillations to optimize visual processing. Accordingly, neurological or psychiatric conditions that perturb either oscillatory dynamics or oculomotor processing likely demonstrate disruptions in theta-oculomotor coupling.

## 9. Parkinson Disease

Parkinson disease (PD), a neurodegenerative disease primarily affecting the basal ganglia, disrupts both oculomotor kinematics and cortical oscillatory activity. For example, PD patients have been shown to exhibit slower, hypometric SEMs followed by prolonged fixations. At the circuit level, the cortico-basal-ganglia loop exhibits excessive beta synchronization (~13–30 Hz) with a reduction in the power and modulation of midfrontal theta and theta–gamma phase–amplitude decoupling [106]. It is these perturbations which are improved with deep brain stimulation (DBS) therapy. Interestingly, this suggests that PD does not only involve motor bradykinesia but a cognitive bradykinesia as well [107]. This shift in oscillatory balance could disrupt the normal theta-band dynamics that underlie flexible behavior. Consistent with this, PD patients show impaired inhibitory control and task switching. For instance, they commit more SEM directional errors with longer latencies in antisaccade paradigms implicating dysfunction in midfrontal theta necessary for cognitive control [107,108].

Converging evidence also indicates that PD disrupts the normal entrainment of oculomotor events and basal ganglia rhythms. In PD patients implanted with DBS electrodes in the subthalamic nucleus (STN), spontaneous eye blinks during walking trigger a delta/theta phase reset in the STN. This is a state-dependent effect that is absent at rest. Interestingly, blink rate, which is tonically low at rest in PD, increases during walking, implicating both network state and dopaminergic tone in how oculomotor events couple to subcortical rhythms in PD [109].

Standard high-frequency STN-DBS (130 Hz) reliably improves motor symptoms in PD but can also alter inhibitory control and decision-making [110,111,112]. It stands to reason that frequency-specific approaches may differentially engage cognitive and motor circuits. Indeed, recent work systematically evaluated three different levels of stimulation delivered to the STN to engage cognitive circuits. In this study, PD patients performed an anti-saccade task under simultaneous eye-tracking and high-density EEG recording with STN-DBS stimulation set at 60 Hz vs. 130 Hz vs. off [113]. Intriguingly, stimulation at mid-frequency (60 Hz) led to increased midfrontal theta power compared to DBS off. This increase in frontal theta power resulted in improved response inhibition. Taken together, these data show that STN stimulation can modulate the oculomotor network in a frequency-dependent manner, with 60 Hz stimulation selectively enhancing frontal theta activity and improving antisaccade performance. In contrast, the clinically standard 130 Hz setting offers motor benefits without the same cognitive effects.

Together, these findings indicate that PD involves under-recruitment of the midfrontal theta network gating goal-directed eye movements, and that frequency-tuned neuromodulation may engage cognitive as well as motor circuits (with theta-oculomotor disruption potentially serving as a biomarker of disease state) [106,108].

## 10. Alzheimer Disease

Alzheimer disease (AD) and related dementias show profound disruptions in oculomotor processing, theta oscillations, and memory encoding. Indeed, in mild cognitive impairment (MCI), an early stage of AD progression, patients are known to exhibit subtle oculomotor processing abnormalities. In the visual paired-comparison (VPC) task, healthy adults preferentially fixate on a novel image longer than a simultaneously presented familiar one. This novelty-driven preference has been linked to intact hippocampal function [102]. However, when the retention interval is lengthened, this novelty preference has been shown to be diminished in MCI patients [114]. A follow-up study demonstrated that VPC performance predicts clinical conversion to AD with lower novelty preference scores. In this study, lower novelty-preference scores identified MCI patients who progressed to AD within ~3 years [115]. Subsequent work refined this into a brief visuospatial memory paradigm which could predict degree of cognitive impairment and progression of disease. Indeed, using eye-tracking metrics as a screening tool has shown promise for early detection of AD with brief gaze tasks able to distinguish healthy patients from MCI/AD by detecting changes in response to visual stimuli. Oculomotor metrics, such as abnormal fixation times on novel images and increased re-fixations on familiar images, may reflect disruption of memory-guided visual exploration. Given the SEM-theta link, these findings suggest disruption of the timing and synchrony of these processes. Further, these data suggest that SEM dynamics and fixation durations can serve as noninvasive biomarkers of early neurodegeneration [116].

Importantly, AD not only alters gaze preference and timing, but also the associated oscillatory activity. In a simultaneous eye-tracking/EEG study, AD patients performing a fixation task exhibited shorter but more frequent fixations compared to age-matched controls. EEG correlates demonstrated increased theta power and reduced alpha power consistent with the well-documented shift in spectral power from higher (alpha/beta) to lower (theta) bands on EEG in AD [117]. Further, parietal alpha activity and fixation events were less tightly coupled suggesting that oculomotor processing is significantly weaker in AD [117]. Hippocampal rhythms are similarly altered with resting-state magnetoencephalography (MEG) revealing increases in theta power with impaired hippocampal-cortical synchrony in prodromal AD. Interestingly, theta power increases can differentiate MCI from age-matched controls [118]. In clinically diagnosed AD, slowing of hippocampal activity is associated with worsened cognitive decline [119]. Notably, in age-matched controls there is a significant correlation between fixation frequency and parietal alpha oscillation power, suggesting that strong alpha rhythms may help stabilize gaze [117].

Thus, the degradation of the normal intrinsic oscillatory activity may decouple oculomotor processing from memory. Early in the disease process, prodromal AD patients may rely on compensatory neural mechanisms during fixations with measurable increases in theta activity and coherence in parieto–occipital networks. However, ultimately this compensatory mechanism fails to regulate oculomotor behavior normally. In more dynamic tasks, AD-related disruption of theta–oculomotor coupling becomes more apparent with AD patients experiencing a failure of internally guided oculomotor behaviors. This is evidenced by a failure to attend to novel details and frequent re-fixations on familiar images.

Taken together, these findings suggest a two-phase pathologic trajectory in AD. First, during prodromal AD, a compensatory theta up-regulation is observed. This is followed by desynchronized, low-energy theta as degeneration advances [120]. Indeed, these studies suggest that eye-tracking metrics and oscillatory activity may be used as biomarkers of disease progression. While this hypothesis remains provisional, concurrent disruption in theta oscillatory and gaze dynamics in a disease marked by attention and memory deficits suggests a strong link.

## 11. Epilepsy

Temporal lobe epilepsy (TLE) can disturb theta oscillations and oculomotor processing in several well-described ways. For example, interictal epileptiform discharges (IEDs) provide an intrinsic perturbation experiment. These brief bursts of abnormal electrical activity are thought to often be clinically “silent” but have been shown to affect hippocampal theta with demonstrable power decreases in the seconds following an IED. Indeed, hippocampal theta may be suppressed for up to 30 s after an IED which abrogates the normal SEM-locked phase-reset window essential for visual input priming [121]. Indeed, a single occipital IED transiently decreases oculomotor processing with slower visual reaction time, stimulus detection and impaired digit-recognition [122]. Further, evidence in rodents demonstrates that hippocampal IEDs during spatial exploration disrupt theta oscillations and degrade spatial working-memory performance [122], supporting a link between IED-mediated theta disruption and visuospatial encoding [123].

Recent work further shows that hippocampal IEDs propagate as travelling waves that disrupt theta-alpha coupling in downstream targets, with closed-loop disruption of these bursts capable of rescuing spatial-memory performance [124,125,126]. Thus, IEDs represent a stochastic and pathologic reset with suppression of local activity capable of de/hyper-synchronizing local oscillatory activity normally imposed by SEM-locked phase reset. Whereas SEM-locked phase-reset is precisely time-locked to a distinct oculomotor event to impart a physiologic and adaptive phase reset across a network, IEDs desynchronize local firing thereby degrading the fidelity of visual sampling and disrupting memory encoding. It stands to reason that inopportunely timed IEDs could disrupt normal oculomotor processing and degrade normal theta-oculomotor synchrony, however, direct evidence for visual processing dysfunction remains limited.

Further, TLE has been found to disrupt normal theta dynamics during visual tasks. For instance, during passive viewing of Ekman faces, patients with TLE showed reduced event-related theta power and phase-locking [127]. In patients with focal epilepsy, resting state MEG revealed weaker hippocampal and medial prefrontal cortex theta coherence with greater decoupling associated with poorer visual memory scores [128]. Similarly, slow theta has been shown to correlate with monthly seizure burden [129]. Robust evidence suggests that oculomotor processing and theta disruption results in network wide dysfunction. Patients with drug-resistant focal epilepsy have been shown to have longer pursuit latencies and an increased number of corrective SEMs during smooth tracking [130]. Further, patients with TLE, during an attention network test, were found to exhibit longer first goal-directed SEM latencies, an increase in the total number of SEMs, and impaired attention [131]. Similarly in children with medication-controlled epilepsy, increased prosaccade latency, poor antisaccade inhibition, was shown to be correlated with slower processing speed and response inhibition deficits [132,133]. Accumulating intracranial evidence also suggests that oculomotor coupling is fragile in TLE. For instance, then an IED occurs within ±500 ms of an SEM, the usual 3–8 Hz phase-reset is blunted and subsequent recall for that fixation is poorer [134]. Depth micro-electrode recordings show that the CD pathway is intact in TLE, but interneuron/principal-cell modulation is diminished in high-spike-rate epochs [98]. Thus, IEDs are associated with desynchronized theta-oculomotor activity, which may underlie reduced visual processing windows through millisecond-to-second reductions in cortical excitability that correlate with impaired reaction times and oculomotor behaviors. This disruption leads to chronic network changes including decreases in theta coherence and slow theta excess which underpins disconnection of hippocampal memory circuits from frontoparietal executive networks. This manifests as inefficient visual search, unstable pursuit, and delayed SEMs. Taken together, a growing body of evidence demonstrates that TLE perturbs the phase alignment of theta with oculomotor movements that normally couples eye movements to visual and memory processing. These data point toward the possibility of rhythm-guided biomarkers, diagnostics and therapeutics coupling.

## 12. Therapeutic Modulation of Theta-Oculomotor Dynamics

The disease-specific perturbations of theta-oculomotor coupling reviewed above suggest that interventions targeting these rhythms may have therapeutic potential. Several approaches have been tested, ranging from noninvasive entrainment to direct stimulation of deep structures. One method of externally driving precise neural oscillatory activity is transcranial alternating current stimulation (tACS). Here, a weak oscillatory current is applied via scalp electrodes to entrain neural networks at a given frequency. This approach has been tested in schizophrenia as an approach to treating the cognitive deficits seen in this disorder. In a single patient, an in-phase 6 Hz tACS was applied to frontoparietal regions during cognitive training with noted improved executive performance and working memory, alongside enhanced midfrontal theta EEG signal after application [135]. However, in a larger study, a 5.5 Hz tACS applied over left frontal or parietal cortex produced no improvements in performance on canonical working-memory tasks despite earlier studies with promising results [136,137,138]. Thus, tACS effects may be subtle or may benefit from individualized or task specific approaches. Given these mixed results, the use of tACS as a therapeutic option remains unclear. Future work might explore closed-loop tACS, where delivered stimulation is contingent on detection of SEM activity specifically reinforcing theta–SEM coupling at precisely defined moments.

The most direct and well-studied modality for modulation of neural circuits is DBS. As we discussed previously, adjusting DBS frequency in PD can engage theta circuits [113]. In AD, DBS of the fornix, a white-matter bundle carrying signals from the hippocampus to basal forebrain, is being explored to slow cognitive decline and memory loss. In the first phase I clinical trial, continuous 130 Hz fornix/hypothalamic stimulation increased temporo-parietal glucose metabolism and decreased cognitive decline in several participants [139]. Subsequent volumetric studies demonstrated reduced hippocampal and mammillary-body atrophy in the same cohort [140]. Building on these observations, small case-series using applied intermittent theta-burst patterns (a 5 Hz, 5-pulse train delivered once per second) with demonstrable enhancements in visuospatial recall in four AD patients [141]. In epilepsy patients, brief, low-intensity theta-burst stimulation of the entorhinal cortex delivered during virtual-navigation learning accelerated route acquisition and improved subsequent spatial recall in medication-refractory epilepsy patients [142]. DBS of the anterior thalamic nucleus (ANT) is a commonly used treatment for drug-resistant focal epilepsy, its efficacy demonstrated in the randomized SANTE trial and sustained at five-year follow-up [143,144]. Anatomically, the ANT is a limbic relay within the Papez circuit, receives dense hippocampal and mammillary inputs, and many of its neurons phase-lock to hippocampal theta oscillations [145,146]. Beyond seizure control, intracranial studies show that low-frequency ANT stimulation can sharpen working-memory precision in humans [147]. Similarly, ANT stimulation in epileptic mice entrains hippocampal rhythms and mitigates seizures [148]. These data suggest that future approaches to DBS stimulation could involve delivering high-frequency trains to abort seizures while periodically giving theta-locked bursts to bolster cognition. Recent proof-of-concept work shows that 4–6 Hz STN stimulation acutely improves working-memory accuracy in patients with PD [149]. A next-generation closed-loop system could exploit this by delivering phase-aligned theta bursts to the STN precisely when eye-tracking detects a goal-directed SEM thereby synchronizing cortico-basal-ganglia loops at the moment executive control is engaged. Such a system would aim not only to ameliorate motor symptoms, as standard high-frequency STN DBS already does, but also to restore the cognitive deficits seen in PD.

Several practical considerations may temper the near-term translation of theta-oculomotor metrics into routine clinical use. High-density EEG is constrained by a labor-intensive setup and the absence of standardized protocols across institutions for use [150]. In PD, parkinsonian tremor generates 4–6 Hz electromyographic artifact that overlaps spatially and temporally with the theta-band signal of interest, complicating artifact rejection even with independent component analysis [151]. Eye-tracking faces constraints with calibration challenges and inter-individual variability which limits reproducibility [152]. These limitations are made worse in patients with dyskinesias or cognitive decline who cannot reliably sustain fixation or follow task instructions [153]. Overcoming these challenges will likely require standardized protocols, simpler or wearable devices, and tasks designed for patients with advanced disease.

## 13. Conclusions

In summary, theta oscillations and SEMs form a tightly coupled system for actively sampling and processing visual information. This system is vulnerable to neurological and psychiatric perturbations. In healthy individuals, SEMs represent an internal pulse that triggers phase reset and cross-regional coherence of theta oscillations. This temporal framework links oculomotor processing with memory encoding. Mechanistically, theta–SEM coupling likely arises from a network involving the hippocampus, FEF, ACC, and midbrain structures including the SC with coordination via thalamic nuclei [102]. When this network functions properly, SEMs and theta oscillations exhibit a bidirectional relationship in which theta phase is associated with the timing and direction of SEMs, while SEMs are accompanied by phase resets that co-occur with enhanced cross-regional coherence. While direct manipulations (e.g., optogenetic SC stimulation, tACS over FEF, frequency-specific DBS) provide causal support for select components of this model, much of the evidence to date remains correlational, and the directionality of these interactions warrants further investigation. Neurological diseases like PD, AD, and epilepsy directly impair neural circuit integrity and neuromodulatory tone perturbing this system. Thus, the study of theta-oculomotor dynamics in neurologic and psychiatric conditions not only allows us to understand the fundamental functions of neural circuits and systems, but it may also potentially open up new avenues for diagnosis and treatment.

## Data Availability

No new data were created or analyzed in this study.
